# Suppression of Menses, with Good Health

**Published:** 1872-04-01

**Authors:** J. A. Hamilton


### SUPPRESSION OF MENSES, WITH GOOD HEALTH.

BY J. A. HAMILTON, M.D.

ODIN, Ill., 1872.

On the 24th day of June, 1868, I was called to see Mrs. I——, a lady eighteen years of age, rather under medium size, dark complexion, hair and eyes, married ten months, never menstruated but once (sparingly), two months previous to her marriage. On my visit I found Mrs. I—— looking tolerable well, up doing her work, as she usually did between her spells each month, when nature would endeavor to establish menstruation, at which time she suffered excruciatingly with her head and back, vascular excitement running very high, almost, and sometimes quite producing convulsions.

This being about two weeks before the expected time for a return of her bad feelings, I saw no necessity for active medication, therefore I administered comp. cathartic pills, to evacuate the contents of the bowels, after which I put her upon mercurial alteratives, the better to prepare her for the administration of medicine directed to the uterine system at a proper time. I kept her on this course of treatment until within three days of her expected time. I then ordered the following:

| | | |
|---|---|---|
| ℞ | Pulvis guaiac, | ℥ i. |
| | Soda et potassa carbon. | gr. xxxi. |
| | Pulvis pimenth. | ʒ ii. |
| | Alcohol, | ℥ iv. |

The vol. sp. ammonia to be added *pro re nata*, teaspoonful to be given three times a day in sherry, tea of rubia tinctorum *ad. lib.*, and hip bath at night. I continued this course of treatment during her suffering this time, which was somewhat mitigated, but without any discharge whatever. I was then told that she had been under the treatment of several physicians before, with the same object in view, and with a like result as myself. I requested an examination per vagina, which was readily acceded to, to see what light might be thrown upon the case. I found the external parts of generation well developed and perfectly natural in every particular, but one and a-half inch from the lobes I found the vagina permanently and persistently occlused, admitting of no passage whatever from the womb. Thus the nature of the case, heretofore enveloped in mystery, now became perfectly plain and apparent, and the remedy unquestionable. I apprised my patient and her friends of the nature of the case, and the means to be used for her relief. My proposition to operate, and divide the walls of the vagina, was not favorably received, and, therefore, of course, I abandoned the case, believing, as I did, there was no other means of relief—informing them, at the same time, I did not think it possible for her to survive many months in her present condition. In this I was very much mistaken. I have watched the case from that time until the present, a period of about three years. The lady is now well and hearty, as much so as any lady of my acquaintance. She tells me that since my visit to her, her trouble has been growing less and less each month, until at present she suffers no more, she thinks, than other ladies do at their monthly periods. Yet there has never been any perceptible discharge in any way whatever. No operation ever having been performed, the vagina still remains closed.

Here is an anomalous case. I have never known anything like it reported, and did not suppose any woman could live, much less enjoy good health, with an entire suspension of so important a function to the female economy, during her menstrual period. It may be that some of my medical friends have seen similar cases, and to such it is not strange ; but to me, I confess I have never seen anything of the kind before, and cannot ever imagine how life can be sustained.

If there were any vicarious discharge to take the place or supersede the necessity of the natural discharge per vagina, which is sometimes the case, then I should not be so much surprised ; but here is a case of perfect health, with an entire suspension, as far as I am able to ascertain, for the space of three years, of the menstrual evacuation.

I have been rather actively engaged in the practice of medicine for about twenty-five years—have never met with anything of the kind before. If there is any such case on record, I have failed to have met with it. I do not know what the medical profession will think or say to such an article as the above ; perhaps, that I am considerable of an egotist, or that I like to see myself in print. I do not think that I am much of an egotist. I am not in the habit of writing for medical papers, or any others. I give the case because it is a rare one, and also because it is an interesting one to the profession—interesting from the fact that it shows how little we know of the powers of nature to accommodate herself to the deviations from the great general laws and regulations which govern us in our being.

This 26th day of March, 1872.

